# Investigation of cytokine imbalance in schizophrenia, assessment of the possible role of serum cytokine levels in predicting treatment response, prognosis and psychotic relapses

**DOI:** 10.1192/j.eurpsy.2024.1425

**Published:** 2024-08-27

**Authors:** K. Bodnár, L. Hermán, R. I. Zsigmond, J. Réthelyi

**Affiliations:** Department of Psychiatry and Psychotherapy, Semmelweis University, 1085, Hungary

## Abstract

**Introduction:**

Schizophrenia, a multisystem chronic psychiatric disorder of unknown etiology, is associated with several immune dysfunctions, including abnormal levels of circulating cytokines. Exsisting evidence shows a potential causative role for cytokines in schizophrenia symptom development. Furthermore, disease duration, symptom severity, aggressive behavior, and cognitive deficits are correlated with levels of certain cytokines. Despite the development of new antipsychotics, the negative and cognitive symptoms of schizophrenia often do not respond adequately to pharmacotherapy.

**Objectives:**

Research questions and hypotheses: 1. Can there be a cytokine or cytokines among the different cytokine levels detected in schizophrenia that can be used as biomarkers of treatment response? 2. Can changes in cytokine levels indicate the occurrence of psychotic relapse? 3. Can changes in the cytokine level play a role in predicting the prognosis of the disease? The secondary objectives of the planned research, in addition to the above, are to clarify the knowledge gathered so far about the relationship between cytokine level changes and the clinical symptoms associated with them.

**Methods:**

We investigate cytokine levels, blood samples are taken on hospital admission. Based on the publications, we mainly focus on the Il-2, Il-4, Il-6 and Il-10 levels, which can serve as possible predictive biomarkers relating to treatment response. We will also assess the possible role of abnormal cytokine levels and their association with symptoms severity and their potential clinical implications. The severity of the symptoms is monitored with the PANSS.

**Results:**

15 schizophrenic patients who were hospitalized due to a psychotic relapse have been included. Blood samples were taken to measure cytokine levels, the PANSS scale was recorded during a psychotic relapse. We have included 9 healthy, age- and gender-matched healthy controls in the study, from whom blood samples were taken to measure cytokine levels. Preparation for measurement of cytokine levels is underway. Patient involvement is ongoing.

**Conclusions:**

A better understanding of cytokine imbalance in schizophrenia patients can potentially help in early diagnosis, novel therapeutic target indentification and development, patient stratification for choosing the best therapeutic protocol, and predicting prognosis, relapse and treatment response.

**Disclosure of Interest:**

None Declared

